# The antigenicity and cholesteroid nature of mycolic acids determined by recombinant chicken antibodies

**DOI:** 10.1371/journal.pone.0200298

**Published:** 2018-08-09

**Authors:** Heena Ranchod, Fortunate Ndlandla, Yolandy Lemmer, Mervyn Beukes, Johann Niebuhr, Juma Al-Dulayymi, Susan Wemmer, Jeanni Fehrsen, Mark Baird, Jan Verschoor

**Affiliations:** 1 Polymers and Composites, Council for Scientific and Industrial Research, Pretoria, South Africa; 2 Department Biochemistry, University of Pretoria, Pretoria, South Africa; 3 School of Chemistry, Bangor University, Wales, United Kingdom; 4 Serology and Immunochemistry, Vaccines and Diagnostics Development Programme, Agricultural Research Council—Onderstepoort Veterinary Institute, Pretoria, South Africa; University of British Columbia, CANADA

## Abstract

Mycolic acids (MA) are major, species-specific lipid components of Mycobacteria and related genera. In *Mycobacterium tuberculosis*, it is made up of alpha-, methoxy- and keto-MA, each with specific biological functions and conformational characteristics. Antibodies in tuberculosis (TB) patient sera respond differently towards the three MA classes and were reported to cross-react with cholesterol. To understand the antigenicity and cholesterol cross-reactivity of MA, we generated three different chicken -derived phage-displayed single-chain variable fragments (scFv) that reacted similarly towards the natural mixture of MA, but the first recognized all three classes of chemically synthetic MAs, the second only the two oxygenated types of MAs and the third only methoxy MA. The cholesterol cross-reactivity was investigated after grafting each of the three scFv types onto two configurations of constant chain domains–CH1-4 and CH2-4. Weak but significant cross-reactivity with cholesterol was found only with CH2-4 versions, notably those two that were also able to recognize the *trans*-keto MA. The cholesteroid nature of mycobacterial mycolic acids therefore seems to be determined by the *trans*-keto MA subclass. The significantly weaker binding to cholesterol in comparison to MA confirms the potential TB diagnostic application of these antibodies.

## Introduction

Tuberculosis (TB) is an infectious disease that is caused by the *Mycobacterium tuberculosis* species of bacteria [1]. Despite this being discovered already for over 100 years the disease continues to cause epidemics worldwide. TB is the leading cause of death due to infectious disease globally, ranking higher than HIV/AIDS according to the latest World Health Organisation report of 2017 [2]. It maintains a heavy burden on economies and human health, not only in the developing countries but also throughout the world. The latest statistics released by WHO in 2017 reported that approximately 1.1 million people were living with TB and HIV co-infection worldwide. The diagnosis of TB is a challenging aspect impacting on the management of the disease. Current TB diagnostic tests have been shown to still exhibit problems including: long time period between testing and accurate diagnosis, not enough sensitivity, not always accurate, and, in some cases, expensive. The 2017 WHO report stated that the diagnostic pipeline is progressing fast enough [2]. TB is a growing epidemic and will expand if the disease is not curbed as soon as possible.

The mycobacterial cell envelope is made up of a variety of antigens of which mycolic acids (MA) represent the major lipid component [3]. They occur either as free acids, linked to glycolipids such as trehalose dimycolate or bound to arabinogalactan of the peptidoglycan layer [4–6]. It is known that TB patients produce anti-mycolic acid antibodies (AMAA). The AMAA levels are maintained in sera of HIV-infected TB patients regardless of a declining CD4^+^ T cell count [7, 8]. This enables a biomarker test based on detection of AMAA to detect active TB disease regardless of the HIV status of the patient, which is often the challenge with antibody (Ab) biomarker tests. Although AMAA are known to exist in TB patients, the antigen moiety of MAs that is recognized by the Abs is not known and the molecular basis that governs MA-specific Ab-MA interactions is not well understood. A well-studied case of lipid antigen recognition by Abs is that of cholesterol, where it has been demonstrated that Ab-lipid interactions do not follow the classical Ab recognition mechanism defined for general proteins. A specific protein antigenic epitope binds to a single Ab specificity, similar to enzyme-substrate recognition, but monoclonal Abs (mAbs) generated against cholesterol recognize the structural arrangement of several cholesterol molecular moieties, rather than a single defined epitope. Monoclonal Abs against cholesterol do not recognize it as a monomeric ligand or hapten, but in its crystalline form, or when in monolayers [9, 10]. Interestingly, a mAb that recognizes cholesterol with the hydroxyl functional group in the 3β-position cannot recognize epicholesterol where the hydroxyl group is in the 3α-position, suggesting different packing of cholesterol molecules in monolayers under different molecular arrangements. Moreover, the same mAb cannot recognize ergosterol that has the same stereochemistry of the hydroxyl functional group as cholesterol [11, 12]. This suggests that the specificity and/or selectivity of these structure-recognizing mAbs depend more on overall structural arrangement of particular steroid molecules than on the specific antibody contact with a spatial orientation of single functional groups of the steroid.

**Fig 1:**
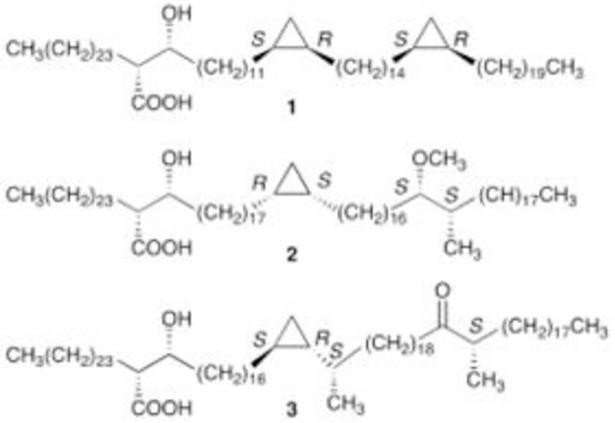
Structures of the three major MA classes from *Mycobacterium tuberculosis*, alpha-MA (1), methoxy-MA (2) and keto-MA (3).

Unlike immobilized cholesterol, which forms from a homogeneous structure, naturally occurring MAs (Fig 1) exist as a chemically heterogeneous mixture of three major classes (alpha-, keto- and methoxy-MA).

These classes are characterized by the presence of chemical functional groups, viz. proximal and distal *cis*-/*trans*-cyclopropane rings for alpha-MA, proximal *cis*-/*trans*-cyclopropane and distal keto for keto-MA and proximal *cis*-/*trans*-cyclopropane and distal methoxy for methoxy-MA [5, 6, 13, 14].

The chemical variations in MA molecule compositions are also present in the cell wall of *M*. *tuberculosis* and differ remarkably between mycobacterial species [5, 6, 13]. Conformational studies of MAs in Langmuir monolayers have shown that the three major classes adopt a four-chain folded conformation that can be seen as a W-shape in two dimensions, with the molecules folding at their proximal and distal functional groups. While keto-MA favored a rigid, fully folded W-shape conformation, alpha- and methoxy-MA had partially folded conformations [15–17]. It was also shown that the folding and packing of MAs are influenced by the classes, with oxygenated MAs containing α-methyl *trans*-cyclopropane groups folding more readily than those with *cis*-cyclopropane rings [18]. This suggests that depending on constituents of natural MAs mixture, the MA antigen will fold differently. The structural relatedness of cholesterol and MAs is supported by (i) the fact that Amphotericin B binds to both MAs and cholesterol, (ii) patient serum Abs produced against MAs cross-react with cholesterol, and (iii) a single mAb against MA was found that binds both cholesterol and MAs [19, 20]. Notably, a mAb against MA was also found that did not cross-react with cholesterol. The structural relatedness between cholesterol and MAs was reportedly responsible for the low accuracy in the serodiagnosis of TB aimed at detecting AMAA using the ELISA assay [8]. It is well established that all humans have anti-cholesterol antibodies (ACHA) in their blood [21], which may in part explain the low Ab activity to MAs in TB negative patients. When designing an AMAA biomarker based test it will be necessary to avoid the detection of cholesterol-binding cross-reactive Abs.

Previously, researchers successfully generated MA-specific mAbs using phage display technology [19, 22]. The rationale for using phage display technology to generate MA-specific mAb is that Abs can be generated against any antigen target, including non-immunogenic or poorly immunogenic antigens such as lipids without the need for immunization [23, 24]. However, in order for mAbs to be used as research tools in diagnostics and in therapeutics, they need to meet certain criteria: (i) be able to bind with adequate affinity and specificity to the target, (ii) be stable and (iii) be readily produced in an affordable expression system such as *E*. *coli* [25]. Although single chain variable Ab fragments (scFv) remain the most used mAb formats [26, 27], they have stability and aggregation problems [28]. A number of different strategies are used to increase scFv stability including Ab engineering after bio-panning/selection and stress-guided selection, i.e. selective pressures such as high temperatures, low pH or high concentrations of guanidinium chloride [25, 29].

The current study was designed to investigate whether the antigenicity and cholesteroid nature of MA is dependent on a particular MA subclass type rather than a combination thereof. To confirm the cholesteroid nature of MAs and to determine the nature of its observed cross-reactivity, a scFv chicken antibody gene library was screened for specific binders to MA and cross-reactivity with cholesterol [30]. The rationale for using chicken antibodies is that, like humans, chickens express specialized MA-presenting CD1 proteins for lipid antigen presentation. It is well-known that lipid antigens can be presented to T cells using CD1 proteins on antigen presenting cells as presenter molecules. Although the CD1 family of molecules was discovered and described in the late 1970s, their function as lipid and glycolipid antigen presenting proteins was only recognized in the early 1990’s, when Beckman *et al*. showed that CD1 proteins present mycobacterial MA on human dendritic cells to CD4/CD8 double negative T cells, causing these to become activated [31]. Mice were found not to have the specific CD1 member that presents MA, but chickens do [32].

**Fig 2:**
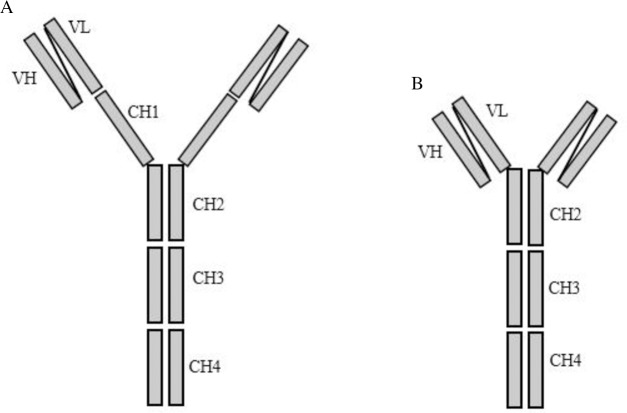
Structures of two types of vectors used for antibody engineering. A: scFvIgY _(CH1-4)_ and B: scFvIgY _(CH2-4)_. Figure adapted from Greunke *et al*. [33].

Three monoclonal chicken scFv Ab fragments with three different specificities were selected: one recognizing all three MA subclasses and cholesterol, another only keto- and methoxy-MA subclasses and a third only the methoxy-MA subclass. In addition, these scFv fragments were engineered into two types of bivalent IgY formats [33] which we refer to as gallibodies, one a theoretically flexible CH1-4 construct and the other a truncated and hypothetically more rigid CH2-4 type as shown in Fig 2.

## Materials and methods

### Screening of the library for MA binders

The naïve semi-synthetic chicken phage-displayed scFv (*Nkuku®*) library containing 2 x 10^9^ phage particles [30] was obtained from ARC-Onderstepoort Veterinary Institute (South Africa). The library contains scFv Ab fragments displayed on recombinant M13 bacteriophage. The scFv Ab fragments were derived from combinatorial pairings of chicken variable heavy chain (V_H_) and variable light chain (V_L_) immunoglobulin domains. V_H_ and V_L_ domains are linked by an interpeptide segment consisting of the sequence (GGGGS)_3_ enabling a fold typical of scFvs. This library was panned as previously described for AMAA [19].

Briefly, Nunc-Maxisorp ELISA plates (Thermo Scientific, USA) were coated with 50 μl of a natural isolated mixture of MAs (Sigma-Aldrich, USA) dissolved in freshly distilled hexane to a final concentration of 250 μg/ml. The coated plates were allowed to evaporate at room temperature and then incubated overnight at 4°C. Plates were washed three times with 300 μl per well of phosphate buffered saline (PBS) containing 0.1% Tween 20. Two blocking agents, 2% Casein (Cas)/PBS pH 7 and 3% bovine serum albumin (BSA)/PBS pH 7 were used alternately during four consecutive rounds of panning to reduce the possibility of enriching for blocking agent-specific binders. Plate wells were blocked with 300 μl of a blocking agent and incubated for 2 h. The *Nkuku*® phage library was pre-incubated with the blocking buffer for 30 min at room temperature and then added to the MA-coated wells and incubated for 2 h at room temperature. Plate wells were then washed twenty times with 300 μl per well of PBS/ 0.1% Tween 20 to remove unbound phages. Stringency of washing was increased during subsequent rounds of panning by increasing the number of washes from three to twenty and simultaneously increasing the wait period between washed from 30 s to 1 min. To elute phages displaying Ab fragments that bound to MA, 150 μl of 100 mM trimethylamine was added to each well and the plate was allowed to shake at (150 rpm) for 10 min. Eluted phage were then neutralised with 150 μl of 1 M Tris, pH 7.4 and used to infect exponentially growing cultures of *E*. *coli* TG1 cells (New England Biolabs Inc., UK). After overnight growth, the bacteria were collected by centrifugation. Phage particles were rescued using M13KO7 helper phage (New England Biolabs Inc., UK), precipitated with PEG/NaCl (20% polyethylene glycol 6000, 2.5 M NaCl) and used as input for the next round of panning. Biopanning was sequentially repeated four times.

### Phage-ELISA

Polyclonal populations of fusion phage produced after each round of selection were tested by ELISA to confirm enrichment of MA-specific phage binders. To identify MA-specific monoclonal phage binders, individual bacterial clones were selected from each round of panning. The MA-specific monoclonal phage binders were further assessed for their long-term storage (four weeks) ability under different temperatures, cross-reactivity with cholesterol and non-specific binding to hydrophobic ligands using the BSA system (previously described; [34]). Nunc-Maxisorp ELISA plates were coated with 50 μl of natural mixture of MAs dissolved in freshly distilled hexane to a final concentration of 250 μg/ml (for polyclonal phage) or 62.5 μg/ml (for monoclonal phage). Cholesterol (Sigma-Aldrich, USA) was dissolved in hexane and coated at both 0.250 mg/ml and 1 mg/ml. Bovine serum albumin was coated at 1 mg/ml. The MA and cholesterol coated plates were allowed to have the hexane evaporate from them at room temperature and were then incubated overnight at 4°C. The following day, plates were washed three times with 300 μl per well of PBS/0.1% Tween 20. The non-specific binding sites were blocked with 300 μl per well of 2% Cas/PBS pH 7 for 2 h whereafter the plates were washed three times with PBS/ 0.1% Tween 20. The PEG precipitated phages were mixed with 2% Cas/PBS-0.2% Tween 20 (1:1, v/v) and 50 μl of this mixture (test solution) was added to each well and incubated for 1 h at 37°C. The test solution was discarded and plates washed three times with 300 μl per well of PBS/ 0.1% Tween 20. A mouse monoclonal anti-M13 mAb B62-FE2 (PROGEN Biotechnik, Germany) was diluted 1:1000 in 2% Cas-PBS/0.1% Tween 20, added to the wells (50 μl per well) and incubated at 30°C for 60 min. The Ab solution was discarded and the plates washed three times with 300 μl of PBS/0.1% Tween 20 per well to remove the unbound Abs. Rabbit anti-mouse IgG conjugated to horseradish peroxidase (HRP) (Sigma Aldrich, USA) was diluted 1:1000 in 2% Cas-PBS/0.1%Tween 20, was then added to each well and incubated at 30°C for 60 min. The Ab solution was discarded and the plate was washed three times with 300 μl PBS/0.1% Tween 20 per well. The signal was developed by adding 50 μl TMB Single Solution Chromogen for ELISA (Invitrogen™, Thermo Scientific, USA) to each well and then incubated at room temperature for 30 min. To stop the reaction, 50 μl of 2 N H_2_SO_4_ was added to each well. Plates were read using a Multiskan Ascent plate reader (Thermo Scientific, USA) set at a measurement of 450 nm and a reference of 620 nm wavelengths.

### Characterization of mAbs binding activities against chemically synthetic MAs

Synthetic, stereo-controlled single MAs were used to test the binding affinity of the phage clones towards the different classes of MAs. The details on their synthesis have been reported [35–42]. The ELISA plates were coated with the different synthetic MAs, namely a 50/50 (v/v) mixture of synthetic MA (alpha- and methoxy-MA or alpha-and keto-MA). Natural MAs served as antigen positive control. The lipids were dissolved in hexane to a final concentration of 62.5 μg/ml and carrier hexane only coated wells served as antigen negative controls. After coating, hexane was allowed to evaporate at room temperature and plates were incubated overnight at 4°C. Phage mAb clones of the same concentration (10^3^ CFU per mL determined by phagemid titre) were tested, using ELISA as described above.

### DNA sequencing

To assess the uniqueness of individual phage clones, the nucleic acids were sequenced on an Applied Biosystems ABI PRISM® 3100 Genetic Analyser (Foster City, USA) using the BigDye® Terminator v3.1 Cycle Sequencing Kit (Applied Biosystems, Foster City, USA), which is based on the dideoxy chain-termination DNA sequencing method of Sanger *et al*., 1997 [43]. Sequence alignment was carried out using BioEdit [44].The following primers were used for amplification of scFv gene inserts from recombinant pHEN1 phagemid vector; forward OP52 primer: 5’ CCCTCATAGGTTAGCGTAACG 3’ and reverse M13rev primer: 5’ CAGGAAACAGCTATGAC 3’ (Inqaba Biotech, South Africa).

### Evaluation of binding as single-chain fragments

Individual clones were grown overnight at 37°C with shaking (220 rpm) in LB medium containing 2% glucose and 100 μg/ml ampicillin. The following day, these cultures were inoculated 1:100 into 50 ml fresh medium containing glucose and ampicillin and grown to midlog (OD_600_ = 0.9). The cultures were then centrifuged and the supernatant fluid (SNF) discarded. Cell pellets were resuspended in 10 ml LB containing ampicillin and 1 mM isopropyl-β-D-thiogalactoside (IPTG) and incubated overnight with shaking at 30°C to induce expression of soluble fragments. Secreted scFv fragments remain in the SNF and were isolated by centrifugation at 3000 rpm for 10 min at 4°C. Cell pellets were retained to harvest periplasmic scFvs by resuspending them in one-tenth volume of 1 × PBS supplemented with 1 M NaCl and 1 mM EDTA and incubating on ice for 30 min. The fractions were centrifuged at 6000 × *g* for 10 min at 4°C and the SNFs containing the scFvs were transferred to fresh tubes. ELISA with MA was conducted to confirm the functionality of the scFvs. Briefly, Nunc-Maxisorp ELISA plates were coated with 50 μl of natural mixture of MAs (Sigma-Aldrich, USA) dissolved in freshly distilled hexane to a final concentration of 250 μg/ml. The coated plates were allowed to have the hexane evaporate from them at room temperature and were then stored until further use. The following day, the ELISA plates were washed three times with 300 μl per well of PBS/0.1% Tween 20. Non-specific binding sites were blocked with 300 μl per well of 4% milk powder/PBS 1 hr at 37°C and then washed three times with PBS/ 0.1% Tween 20. Isolated scFvs were diluted with 2% milk powder/PBS-0.05% Tween 20 (1:1, v/v) and 50 μl of this mixture was added to each well. Plates were incubated for 1 h at 37°C. The test solution was discarded and plates washed three times with 300 μl per well of PBS/ 0.1% Tween 20. Mouse anti-c-*myc* tag mAb 9E10 (Onderstepoort, South Africa) diluted 1:1 in 4% milk powder-PBS/0.1% Tween 20 was added to the wells (50 μl per well) and the plates incubated at 37°C for 1 h. The Ab solution was discarded and the plates washed three times with 300 μl of PBS/0.1% Tween 20 per well to remove any unbound Abs. Polyclonal rabbit anti-mouse immunoglobulin (Dako, Denmark) diluted 1:1000 in 2% milk powder/PBS-0.05% Tween20 was added to the wells (50 μl per well) and the plates incubated at 37°C for 1 h. The conjugate solution was discarded and the plates washed three times with 300 μl of PBS/0.1% Tween 20 per well. The signal was developed by adding 50 μl of TMB Single Solution Chromogen for ELISA (Invitrogen™, Thermo Scientific, USA) to each well and then incubated at room temperature for 15 min. To stop the reaction, 50 μl of 2 N H_2_SO_4_ was added to each well. Plates were read at 450 nm (Thermo Electron Corporation Multiskan EX plate reader).

### Antibody engineering

Single chain variable fragments antibody inserts were prepared for cloning following methods previously described [45]. Briefly, scFv inserts were amplified from plasmid templates by PCR using primers to introduce a *BsiW* (5’ GATCCGTACGGCCGTGACGTTGGACG 3’) and an *AscI* (5’ GATCGGCGCGCCACCTAGGACGGTCAGGG 3’) cleavage site (Inqaba Biotech, South Africa). Restriction digests were performed on scFv inserts (Anti-MA 12, 16 and 18) and IgY-format expression vectors (scFvIgY _(CH1-4)_ and scFvIgY _(CH2-4)_) [33] prior to sub cloning. Ligated plasmids were transformed into JM109 chemically competent *E*. *coli* (Promega, Madison, USA) and amplified under ampicillin resistance. Five clones from each ligation was picked for colony PCR using the forward primer (5’ TAATACGACTCACTATAGGG 3’) and reverse primer (5’ AGGAGGAGGGGTGGAGGACC 3’) to check for the presence of the scFv inserts. Sequences obtained from analysis (Inqaba Biotech, South Africa) of plasmid DNA were compared to the original templates using BioEdit [44].

### Gallibody production and purification

Human embryonic kidney (HEK) 293-H cells (Invitrogen™, Carlsbad, USA) were grown to a confluency of 80–100% in Dulbecco’s Modified Eagle Medium (DMEM)(ThermoFischer Scientific, USA), supplemented with 10% (v/v) foetal bovine serum (FBS) and transfected with 2.5 μg plasmid DNA using TransIT®-293 Transfection Reagent (Mirus Bio Products, Madison, USA). Cultures were grown at 37°C and 5% CO_2_ gas. Successfully transfected cells continued to grow under antibiotic selection (Zeocin ™, Invitrogen™, Carlsbad, USA) and were expanded in fresh tissue culture flasks containing DMEM, 10% FBS and > 50 μg/ml Zeocin. Gallibodies were purified from cell culture supernatants using nickel-nitrilotriacetic acid agarose according to the manufacturer’s (QIAGEN®, Hilden, Germany) instructions. Purity and specificity of the purified gallibodies was checked using SDS-PAGE (See S2 Fig), western blot and ELISA. Purified gallibodies were concentrated in spin columns with a concurrent buffer change (0.1 M borate buffer, pH 7.4) using Vivaspin 10 000 MW PES centrifugation columns (VivaScience, Satorius Group, United Kingdom).

### Gallibody ELISA

Nunc-Maxisorp ELISA plates were coated with 50 μl of natural mixture of MAs (Sigma-Aldrich, USA) dissolved in freshly distilled hexane to a final concentration of 250 μg/ml. Cholesterol was dissolved in hexane and coated at 1 mg/ml. The coated plates were allowed to have the hexane evaporate from them at room temperature and were then stored until further use. The following day, the ELISA plates were washed three times with 300 μl per well of PBS/0.1% Tween 20. Non-specific binding sites were blocked with 300 μl per well of 2% Cas/PBS pH 7 for 2 hrs at 37°C and then washed three times with PBS/ 0.1% Tween 20. Purified gallibodies were diluted to 1 mg/ml with 2% Cas/PBS-0.2% Tween 20 (1:1, v/v) and 50 μl of this mixture was added to each well. Two-fold serial dilutions of each gallibody were prepared on the antigen coated plates, with the concentration range varying from 2 μg/ml to 1000 μg/ml. Plates were incubated for 1 h at 37°C. The test solution was discarded and plates washed three times with 300 μl per well of PBS/ 0.1% Tween 20. Goat anti-chicken Fc: HRP (AbD Serotec, Kidlington, UK) diluted 1:1000 in 2% Cas-PBS/0.1% Tween 20 was added to the wells (50 μl per well) and the plates incubated at 30°C for 1 h. The Ab solution was discarded and the plates washed three times with 300 μl of PBS/0.1% Tween 20 per well to remove any unbound Abs. The signal was developed by adding 50 μl of TMB Single Solution Chromogen for ELISA (Invitrogen™, Thermo Scientific, USA) to each well and then incubated at room temperature for 5 min on MA and 20 min on cholesterol, respectively. To stop the reaction, 50 μl of 2 N H_2_SO_4_ was added to each well. Plates were read at 450 nm (Thermo Electron Corporation Multiskan EX plate reader).

### Statistical analyses

In order to normalize antibody binding signals between different plates, the ratio differences of antibody binding signals relative to casein blocked non-antigen coated wells was determined and data adjusted accordingly. Data analyses was performed using the student’s t-test at a confidence level of 95%.

## Results

### Affinity enrichment of chicken antigen specific phage binders by bio-panning

To evaluate fusion phage specificity, individual clones were selected from the pooled outputs of all four rounds of panning. Only phage binders that retained reactivity to MA after 1 week of storage at 4°C were used as a pool for selecting stably expressed monoclonal phage binders [25]. The next step was to distinguish true binders from false binders in the stable pool of phage binders. Approximately 270 individual clones were screened by phage-ELISA assay. Although a large number of unspecific phages remained after numerous panning rounds, these were reduced to five which were shown to be stable after one week of incubation at 4°C [46]. Two bound specifically to MA, another reacted with both MA and cholesterol and the remaining bound non-specifically.

### Cholesterol and MA cross-reactive phage-displayed scFv mAb

**Fig 3:**
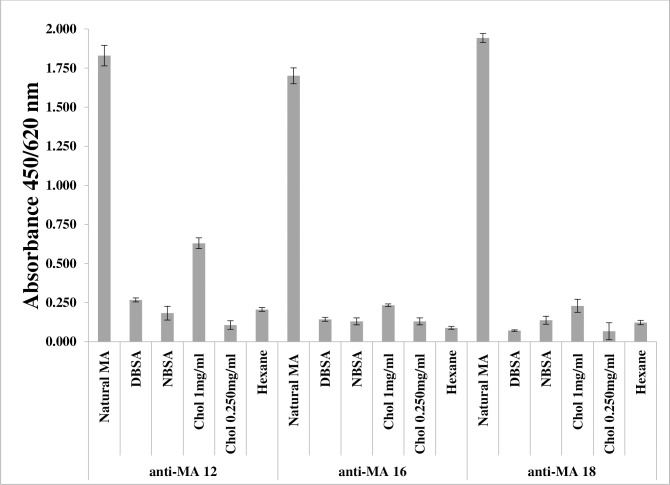
Evaluation of the selectivity and specificity of MA-reactive fusion-phage clones in ELISA. Natural MA was used as a positive control, while denatured bovine serum albumin (DBSA), native bovine serum albumin (NBSA) and hexane were used as negative controls. MAs were coated at 0.250 mg/ml while cholesterol was coated at both 0.250 mg/ml and 1 mg/ml. The concentration of bovine serum albumin used was 1 mg/ml. Error bars = Standard error of mean, n = 5 (biological repeats). See S1 Dataset.

The specificities of the three MA reactive phage clones were further evaluated by ELISA. Denatured BSA system was used with exposed hydrophobic patches that are hidden in native BSA [34]. If the monoclonal phage-fused scFv Abs are not specific to MA, it is expected that they would also bind to hydrophobic patches of BSA. Our results show that anti-MA 12 reacted with both cholesterol (at the higher concentration of 1 mg/ml) and MAs. The binding to these molecules is selective, as anti-MA 12 did not bind to either native BSA (NBSA) or denatured BSA (DBSA) through hydrophobic interactions. Anti-MA 16 and anti-MA 18 showed slight binding to cholesterol only at the higher concentration (p < 0.005) but were not reactive to BSA (Fig 3).

### MA-specific phage-displayed scFvs have different fine specificities of MA binding

**Fig 4:**
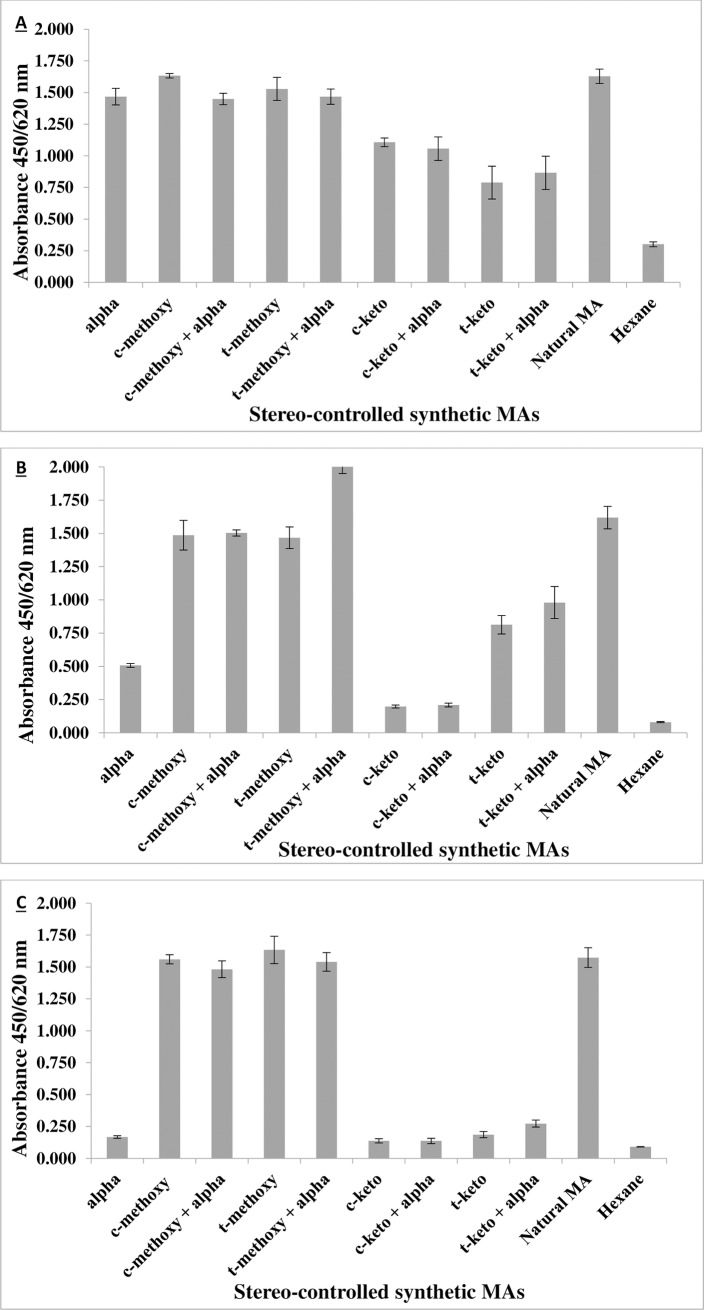
Characterization of the binding specificities of MA-specific phage-displayed Abs with ELISA using single stereo-isomers of stereo-controlled synthetic MAs, their 1:1 mixtures with α-MA and natural MA mixture. In this monoclonal phage ELISA assay, the coating of MA antigens was done by adding 50 μl per well of MA (62.5 μg/ml) dissolved in hexane. Hexane alone was used as a negative control. A) Anti-MA 12, B) Anti-MA 16, C) Anti-MA 18. Error bars = Standard error of mean, n = 8 (biological repeats) t = *trans*, c = *cis*. The experiments on all synthetic MAs were performed with the three mAbs in parallel under the same conditions. This result is a representative of more than three biological repeats with six technical repeats for each mAb. See S1 Dataset.

The fine specificities of the isolated mAbs to MAs were then investigated using stereo-controlled chemically synthetic MA classes (alpha-, keto- and methoxy-) in ELISA (Fig 4).

Anti-MA 12 recognized all classes of MAs. Anti-MA 16 reacted with all classes of MAs except *cis*-keto. Anti-MA 18 bound strongly and exclusively to methoxy MA of either *cis*- or *trans*- configuration and irrespective of whether associated with 50% alpha-MA composition. Alpha-MA did not influence the antigenic properties of MA, since a combination of either keto- or methoxy- with alpha-MA did not result in reduced or enhanced recognition of MA antigen by the selected mAbs.

### Amino acid sequences of the hypervariable regions of the MA-specific phage-displayed scFvs

Since anti-MA 12, 16 and 18 were shown to bind with three unique specificities, the Abs were sequenced to test whether their amino acid sequences are also different. The alignment (Table 1) showed the expected homology between the three monoclonal scFv Abs for the framework regions as reported in literature [30], while the complementarity-determining regions (CDRs) differed.

**Table 1:** Deduced amino acid sequences of CDR 3 regions for phage displayed monoclonal scFv Abs isolated against MA.

Clone	V_H_ CDR3	V_L_ CDR3
Anti-MA mAb 12	MNYRRRQ	TEDSTY
Anti-MA mAb 16	RRITNK	RDSGAP
Anti-MA mAb 18	RKTNKHRIDAWGHGTEV	GSYEASNSAGIFG

V_H_ variable heavy chain, V_L_ variable light chain

It is known that the heavy chain CDR3 plays the dominant role in the observed binding specificity of Abs. The specificity conferred by the heavy chain is preserved regardless of its pairing with various light chains [47]. The CDR1 and CDR2 regions of both heavy and light chains showed minimal sequence diversity, but the CDR 3 regions of both heavy and light chains confirmed that the three monoclonal scFv Abs are unique (Table 1). The most striking result was that the anti-MA mAb 18 showed significantly longer CDR3 regions in both the heavy and the light chain. Whereas CDR1 and CDR2 are known to come about by point mutation only, the larger CD3 additionally comes about by rearrangement of the V genes with D genes (H- chain) and J genes (H- and L- chains) with intermittent random nucleotide insertions.

### Mycolic acid specific monoclonal gallibodies

The clones produced during the antibody engineering process were sequenced to determine the success of the subcloning procedure. Three of the newly generated clones for each of the two gallibody constant chain frames (CH1-4 and CH2-4) were selected for the subsequent gallibody production (see S1 Fig).

To confirm the specificity of the monoclonal antibodies following the conversion from phage-displayed scFvs to purified monoclonal IgY-like antibodies (See S2 Fig), gallibodies were analysed with ELISA on MA. Samples were loaded at a starting concentration of 1000 μg/ml of gallibody and titrated down to 2 μg/ml on the antigen coated plates.

**Fig 5:**
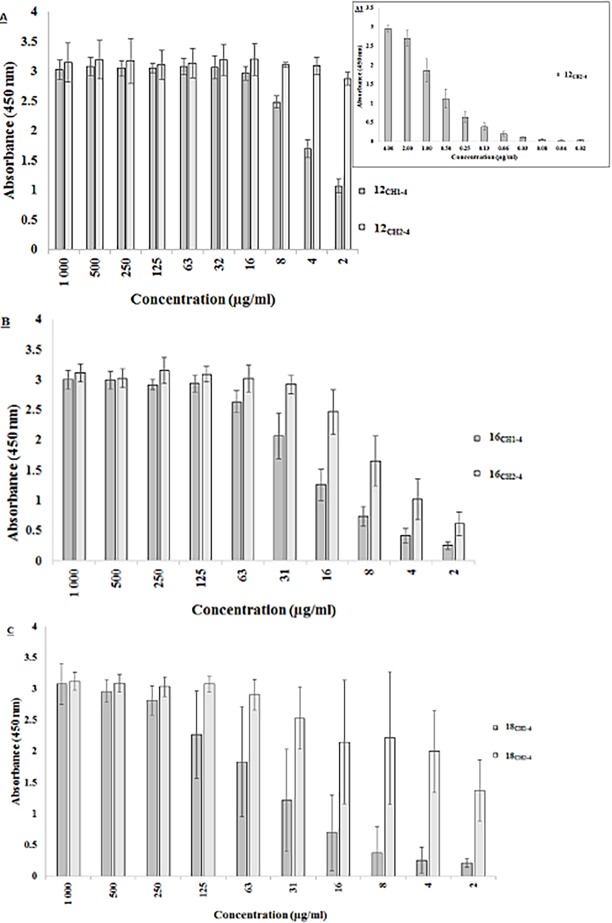
Gallibody binding to natural MAs in ELISA. **Coating of MA antigens was done at 250** μ**g/ml in hexane.** Hexane alone was used as a negative antigen control for background correction of the data. A) Anti-MA 12, B) Anti-MA 16, C) Anti-MA 18. Error bars = Standard deviation, n = 8 (biological repeats), CH1-4 = full length constant region, CH2-4 = truncated constant region. This result is representative of two biological repeats with four technical repeats for each concentration of gallibody. Insert A1) represents gallibody 12_CH2-4_ titration at lower concentrations. No non-specific binding of gallibodies was observed when the antigen negative control was used. See S2 Dataset.

Fig 5A shows that gallibody 12_CH2-4_ was able to bind MA across the entire concentration range, with the amount of binding remaining the same even when an antibody concentration as low as 2 μg/ml was used. The sensitivity for gallibody 12CH2-4 was reached when the antibody was used at a concentration of 1 μg/ml (Fig 5A1). The sensitivity for gallibody 12_CH1-4_ on MA was weaker at A_50%_ = 4 μg/ml. A_50%_ was determined by identifying the concentration of gallibody where absorbance was approximately 50% of the highest signal.

Gallibody 16 showed a slightly weaker sensitivity of MA binding at A_50%_ of 25 μg/ml and 8 μg/ml for gallibody 16_CH1-4_ and 16_CH2-4_, respectively. Gallibody 18 showed the weakest binding to MA with A_50%_ for gallibody18_CH1-4_ at 75 μg/ml and gallibody 18_CH2-4_ at 16 μg/ml.

### Nature of cholesterol cross-reactivity

**Fig 6:**
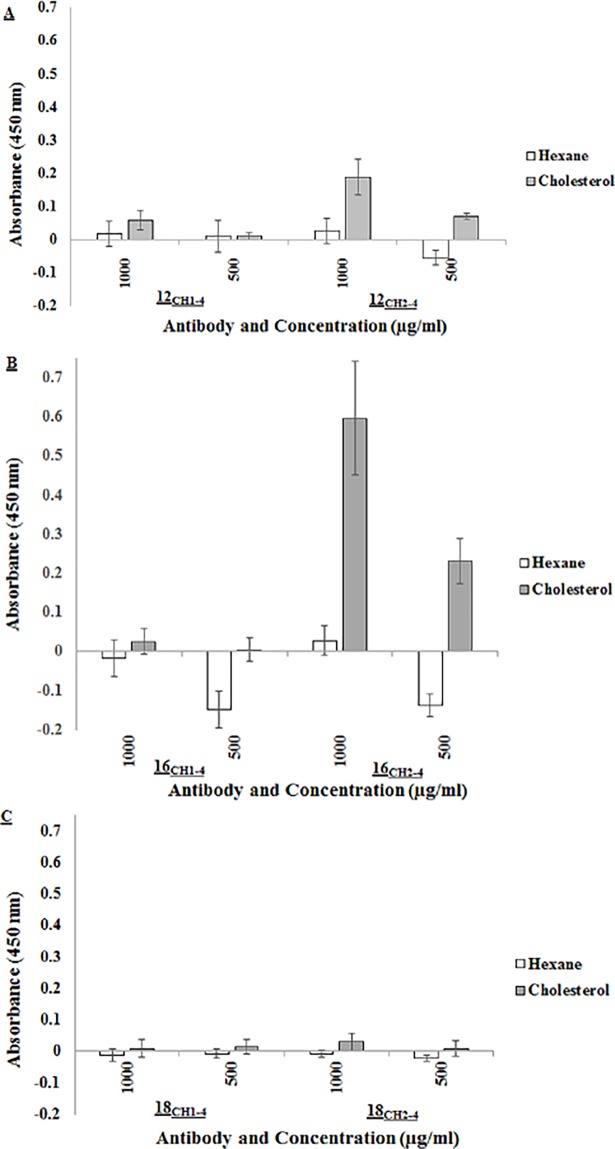
Cholesterol cross-reactivity of gallibodies confirmed with ELISA using hexane solvent alone as negative control. Coating of cholesterol was done at 1 mg/ml in hexane. A) Anti-MA 12, B) Anti-MA 16, C) Anti-MA 18. Error bars = Standard deviation, n = 8 (biological repeats) CH1-4 = full length constant region, CH2-4 = truncated constant region. The results each represent two biological repeats with four technical repeats for each concentration of gallibody. The data were extracted from the same experiment that was performed in Fig 5, making the absorbance readings from both figures directly comparable. See S3 Dataset.

In an attempt to probe the cholesterol cross-reactivity of MA, the gallibodies’ ability to bind to cholesterol was analysed with ELISA. Fig 6 illustrates the cholesterol cross-reactivity for all the gallibodies.

The results show that all of the CH1-4 gallibody types (12_CH1-4_, 16_CH1-4_ and 18_CH1-4_) and one of the CH2-4 type (18_CH2-4_) did not produce signals that were significantly different from the antigen negative control, indicating that these antibodies were not able to cross-react with cholesterol. In contrast, the truncated gallibody 12_CH2-4_ and 16_CH2-4_ cross-reacted with cholesterol. The binding signal of gallibody 16_CH2-4_ was more than two fold higher than the 12_CH2-4_ version, suggesting that the former has a higher affinity for cholesterol.

## Discussion

Our group previously demonstrated that it is feasible to generate MA-specific recombinant scFv antibody fragments from a semi-synthetic chicken antibody-gene library [19]. We used the approach that Beukes *et al*., (2010) used previously to pan for MA-specific Abs from the recombinant chicken antibody library, but selected for thermally stable scFvs. Similarly to Beukes and colleagues (2010), both cholesterol cross-reactive and non-cross-reactive anti- MA phage antibodies were isolated [19].

It appeared as if all the anti-MA phage antibodies recognised cholesterol, with anti-MA 12 giving the highest binding. The binding to cholesterol was much weaker than the binding to MA. We confirmed the specificity of cholesterol binding by using denatured BSA as a negative antigen control. The phage-displayed antibodies showed increased binding to a higher concentration of cholesterol, confirming that cholesterol is more antigenic at the higher concentration as reported by other authors [10].

While the method of phage display technology is useful for selecting scFvs with different binding specificities, it has been reported that soluble scFvs have the ability to naturally form dimers and trimers [48, 49], forming multivalent non-covalently bound antibody fragments. This may give false-positive cross-reactive binding due to the uncontrolled valency for antigen binding. While the soluble scFvs retained their ability to bind to MA, the data obtained revealed their low binding affinity (data not shown). Thus in order to truly define the binding specificities of the selected mAbs, the three scFvs were subcloned into bivalent IgY expression vectors. Results of the gallibody characterization were in agreement with those obtained with phage-displayed Abs, illustrating that all gallibodies bound to MA with approximately equal strength. Cholesterol binding results revealed that only the CH2-4 gallibody subtypes of anti-MA 12 and anti-MA 16 bound to cholesterol at high antibody concentrations. Furthermore, in contrast to the results obtained with phage antibodies, where phage antibody 12 bound strongest to cholesterol, gallibody 16_CH2-4_ now displayed the highest binding. This emphasizes the importance of controlling both protein concentration and antigen binding valency for quantitatively comparing cross-reactivity of anti-MA antibodies. Gallibody 18_CH2-4_ qualified the result found with phage antibody 18 in that it showed no affinity of binding for cholesterol. Methoxy MA is known to be the most antigenic for sensitive detection of anti-MA detection in TB patients [19, 22]. Anti-MA 18, which recognizes methoxy-MA but not keto-MA, has a very unique and long CDR3 region compared to anti-MA12 and anti-MA 16. The CDR3 region is the main carrier of diversity and specificity in the Ab binding site. Other researchers have shown that not only are the differences in sequence of the CDR3 heavy chains between Abs important in determining specificities but also the length of CDR3 heavy chains [50].

In a study by Chan and colleagues (2013) aimed at generating anti-MA Abs from a human antibody gene library using phage-display technology, the authors reported that they could not find a cholesterol cross-reactive anti-MA Ab [22]. This observation is corroborated by our findings of all three CH1-4 gallibodies. However, cross-reactivity with cholesterol was observed with gallibody 12_CH2-4_ and gallibody 16_CH2-4_ at high antibody concentrations (1000 μg/ml and 500μg/ml). Gallibody 12 and 16 both have specificity for binding to *trans*-keto MA as such or in combination with alpha- or methoxy- MAs. In the study of Chan *et al*., all anti-MA Ab generated could not recognize keto-MA but had weak binding to alpha-MA. Our study corroborates this finding as the 18_CH2-4_ gallibody–which also does not recognize keto MA–similarly did not cross-react with cholesterol. Anti-MA 18 only recognizes methoxy mycolate as such or in combination with alpha-MA (Fig 4). Anti-MA 16 recognizes all the synthetic MAs and combinations thereof, except *cis*-keto MA (Fig 4). Notably the lack of specificity of anti-MA 16 towards binding of *cis*-keto MA had no influence on the cholesterol cross-reactivity. Due to these findings, it seems likely that the cholesteroid nature of the MA antigen manifests in the fine specificity towards the *trans*-keto MA subtype.

Interestingly, an involvement of keto MA with cholesterol *in vivo* has recently been reported by Vermeulen *et al*. [51]. These authors wanted to determine the effect that the different MA subclasses have on the growth of the mycobacteria in macrophages, particularly the accumulation of cholesterol brought about by the *M*. *tuberculosis* organism during disease progression. Their results revealed that the keto MA is responsible for the accumulation of intracellular lipid droplets packed with cholesterol. Furthermore, when mouse macrophages were treated with keto MA, bacterial growth was largely unrestricted compared to those treated with alpha- or methoxy- MA.

Notably, from the results obtained it was only selective CH2-4 gallibody types that displayed cholesterol cross-reactivity. None of the CH1-4 gallibodies possessed this ability. This finding can be related to the structures of the two IgY framework constructs used for antibody engineering. The IgY immunoglobulin class of antibodies has been described in the literature as the avian population’s equivalent of the IgG subclass. While these antibodies are similar in structure, one of the most notable differences is the IgY subclass’ lack of a hinge region [52]. These antibodies (IgY) rely on their CH1 and CH2 domains for flexibility, with the CH1 domain contributing largely in this respect [53]. As a result of this and the fact that the CH2-4 construct lacks the CH1 domain, one may refer to the two gallibody subtypes as the flexible CH1-4 and the rigid truncated CH2-4. The CH2-4 versions of the three antibody specificities were generally more sensitive for antigen binding than the CH1-4 versions. This may explain why only the rigid CH2-4 anti-MA monoclonal antibodies cross-react to cholesterol. It is probably due to divalent binding to repeating cholesteroid epitopes on clusters of immobilized MA, which is enhanced by better positive cooperative binding of the two more closely cropped Fab domains on CH2-4 antibody constructs. Mathebula *et al*. (2009) previously showed how mycolic acids cluster into small islands on a self-assembled monolayer of octadecane thiol [54]. The cholesteroid epitopes in the clusters may be too close, and the MA clusters too far apart to be recognized divalently by the more flexible and larger CH1-4 type of gallibody. Thus the binding of the two CH2-4 gallibodies is probably due to the enhanced co-cooperativity aiding in the sensitivity.

The weak affinity of these recombinant mAbs relates to the binding activity of natural antibodies to MA found in TB patient sera. Previously research conducted within our research group demonstrated this property of AMAA–their low affinity. While these antibodies can be detected using ELISA, we revealed how cases can be missed when this standard technique is used for detection [8, 55]. As a result of this more sensitive biosensor techniques for detecting these AMAA, are applied for the diagnosis of TB patients [7].

It has been shown before that the three MA major classes (alpha-, keto- and methoxy-MA) of *M*. *tuberculosis* have varying antigenicity with respect to antibody recognition and that cross-reactive antibodies binding to both MA and cholesterol exist in human sera. This has now been investigated using recombinant anti-MA mAbs derived from a recombinant chicken Ab repertoire. The major findings of this study substantiate the dominant antigenic nature of methoxy-MA reported before, while now demonstrating that the cholesteroid nature of MAs seems to depend on the ability of the AMAA to recognize the *trans*-keto MA subtype.

## Supporting information

S1 Fig Sequences of gallibody clones produced by antibody engineering. 12) Anti-MA 12, 16) Anti-MA 16, 18) Anti-MA 18, CH1-4 = full length constant region, CH2-4 = truncated constant region, V_H_ = variable heavy chain, V_L_ = variable light chain.
(TIF)

S2 Fig SDS-PAGE analysis illustrating gallibody purification using Ni-NTA affinity columns. A) 12_CH1-4_, B) 16_CH1-4_, C) 18_CH1-4_, D) 12_CH2-4_, E) 16_CH2-4_, F) 18_CH2-4._ Gel lanes 1) Marker, 2) Culture supernatant, 3) Flow through 1, 4) Flow through 2, 5) Washes, 6) Elution 1, 7) Elution 2, 8) Elution 3, 9) Elution 4. Successful purification is demonstrated by the comparable thickness of the 67 kDa band obtained with the culture supernatant (2) and the elutions (6–9).
(TIF)

S1 Dataset Experimental data used for producing Figs 3 and 4. (XLSX)

S2 Dataset Experimental data used for producing Fig 5. (XLSX)

S3 Dataset Experimental data used for producing Fig 6. (XLSX)

## Abbreviations

MA: mycolic acids
TB: tuberculosis
scFv: single chain variable fragment
AMAA: anti-mycolic acid antibodies
mAb: monoclonal antibody
ACHA: anti-cholesterol antibodies
E. coli: *Escherichia coli*
CD: cluster domain
V_H_: variable heavy chain
V_L_: variable light chain
Cas: casein
IPTG: isopropyl-β-D-thiogalactoside
SNF: supernatant fluid
NBSA: native BSA
DBSA: denatured BSA
CDR: complementarity-determining regions
