# Supplementary figures and images for: The antigenicity and cholesteroid nature of mycolic acids determined by recombinant chicken antibodies
Source: PLoS One. 2018 Aug 9;13(8):e0200298. doi: 10.1371/journal.pone.0200298 (PMC6084858; doi:10.1371/journal.pone.0200298)

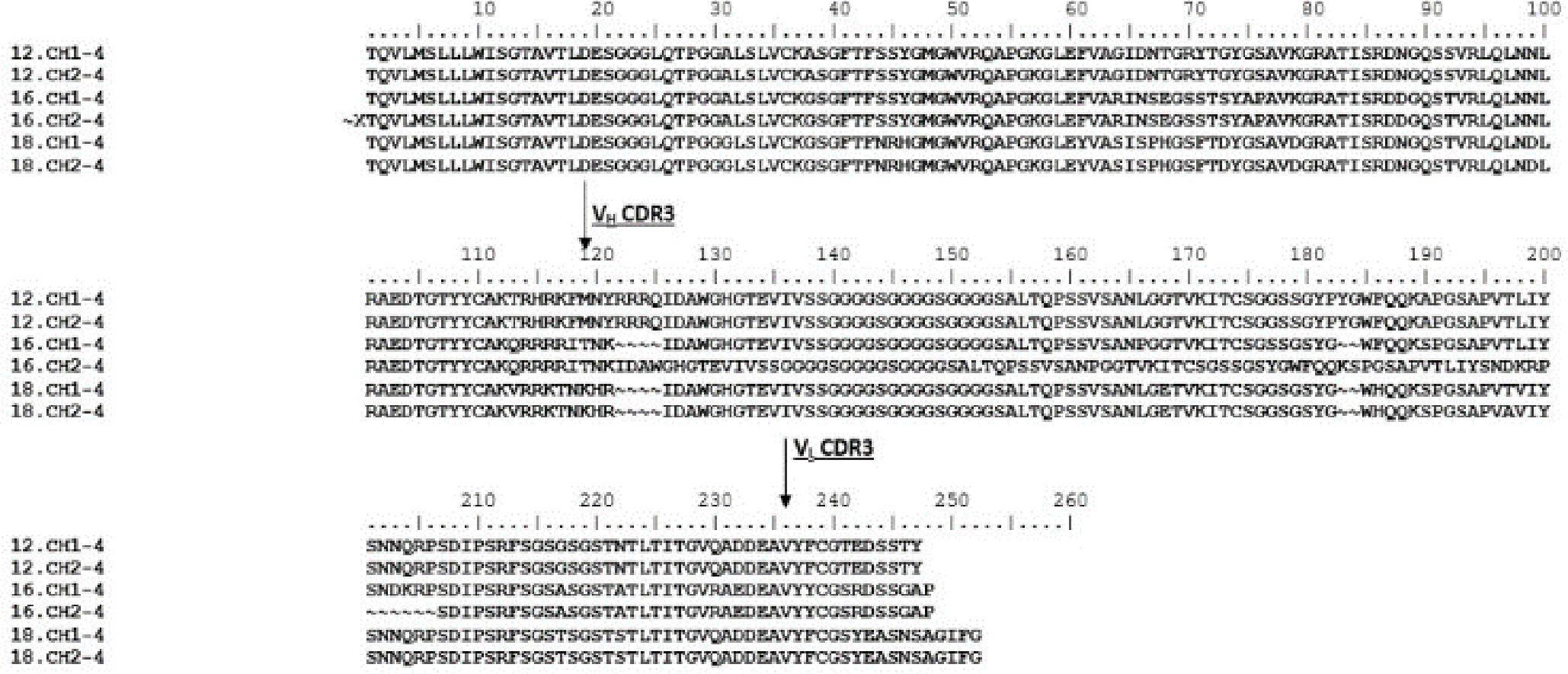

Supplement: S1 Fig — 12) Anti-MA 12, 16) Anti-MA 16, 18) Anti-MA 18, CH1-4 = full length constant region, CH2-4 = truncated constant region, VH = variable heavy chain, VL = variable light chain. (TIF) [file pone.0200298.s001.tif]

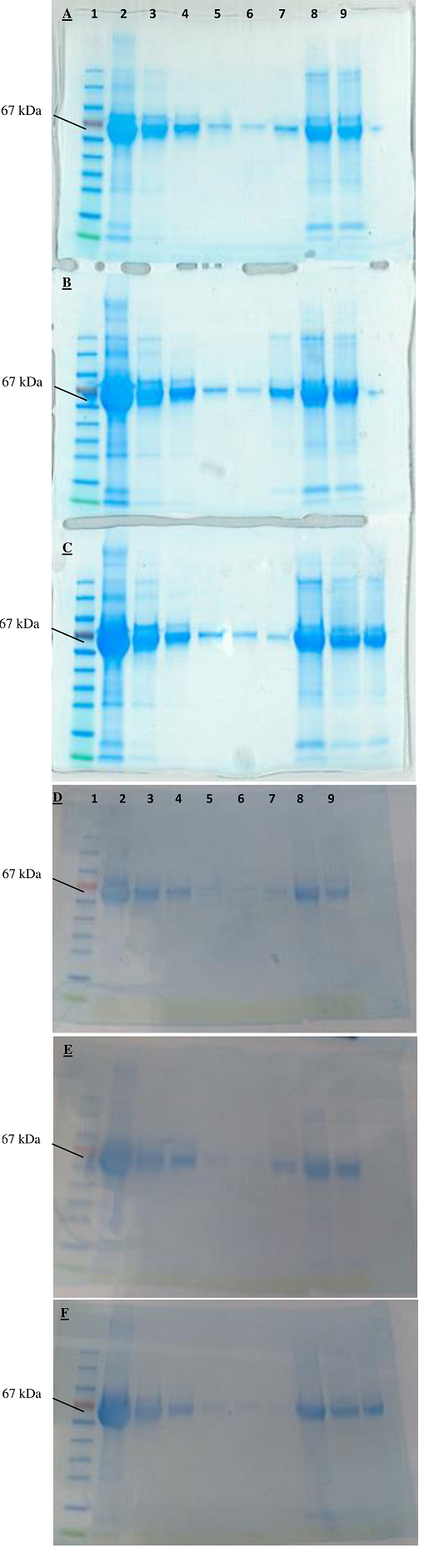

Supplement: S2 Fig — A) 12CH1-4, B) 16CH1-4, C) 18CH1-4, D) 12CH2-4, E) 16CH2-4, F) 18CH2-4. Gel lanes 1) Marker, 2) Culture supernatant, 3) Flow through 1, 4) Flow through 2, 5) Washes, 6) Elution 1, 7) Elution 2, 8) Elution 3, 9) Elution 4. Successful purification is demonstrated by the comparable thickness of the 67 kDa band obtained with the culture supernatant (2) and the elutions (6–9). (TIF) [file pone.0200298.s002.tif]
